# Lycopene and Vascular Health

**DOI:** 10.3389/fphar.2018.00521

**Published:** 2018-05-23

**Authors:** Ioana Mozos, Dana Stoian, Alexandru Caraba, Clemens Malainer, Jarosław O. Horbańczuk, Atanas G. Atanasov

**Affiliations:** ^1^Department of Functional Sciences, “Victor Babes” University of Medicine and Pharmacy, Timiṣoara, Romania; ^2^Center for Translational Research and Systems Medicine, “Victor Babes” University of Medicine and Pharmacy, Timiṣoara, Romania; ^3^2nd Department of Internal Medicine, “Victor Babes” University of Medicine and Pharmacy, Timiṣoara, Romania; ^4^1st Department of Internal Medicine, “Victor Babes” University of Medicine and Pharmacy, Timiṣoara, Romania; ^5^Independent Researcher, Vienna, Austria; ^6^Institute of Genetics and Animal Breeding, Polish Academy of Sciences, Magdalenka, Poland; ^7^Department of Pharmacognosy, Faculty of Life Sciences, University of Vienna, Vienna, Austria

**Keywords:** lycopene, arterial stiffness, endothelial function, intima-media thickness, cardiovascular risk

## Abstract

Lycopene is a lipophilic, unsaturated carotenoid, found in red-colored fruits and vegetables, including tomatoes, watermelon, papaya, red grapefruits, and guava. The present work provides an up to date overview of mechanisms linking lycopene in the human diet and vascular changes, considering epidemiological data, clinical studies, and experimental data. Lycopene may improve vascular function and contributes to the primary and secondary prevention of cardiovascular disorders. The main activity profile of lycopene includes antiatherosclerotic, antioxidant, anti-inflammatory, antihypertensive, antiplatelet, anti-apoptotic, and protective endothelial effects, the ability to improve the metabolic profile, and reduce arterial stiffness. In this context, lycopene has been shown in numerous studies to exert a favorable effect in patients with subclinical atherosclerosis, metabolic syndrome, hypertension, peripheral vascular disease, stroke and several other cardiovascular disorders, although the obtained results are sometimes inconsistent, which warrants further studies focusing on its bioactivity.

## Introduction

Cardiovascular disorders (CVD) are the leading mortality cause worldwide and prophylactic measures to combat it deserve special attention. Atherosclerosis is, in most cases, the main manifestation of CVD, and its progression may be clinical silent for a long time, up to a certain moment, when it directly leads into a severe adverse event (e.g., heart attack or stroke). Beneficial lifestyle changes are important and most cost-effective components of prevention or treatment of cardiovascular disorders. Compounds occurring in plants often display diverse bioactivities with therapeutic potential and different plants and plant-derived compounds have a long history of being reported to exhibit effects counteracting CVD (Atanasov et al., 2015; Waltenberger et al., 2016). Among lifestyle factors, a healthy diet is considered a cornerstone of cardiovascular disease prevention, and the inclusion of sufficient fruits and vegetables in the diet is regarded as especially important (Böhm, 2012; Piepoli et al., 2016). The prevalence of cardiovascular disorders is remarkably unevenly distributed in developed countries, and some areas, e.g., Southern Europe, seem to be protected by having significantly less prevalence of the disease. This effect has often been attributed to dietary factors, as e.g., the Mediterranean diet, with a lot of vegetables, including tomatoes, tomato products, and olive oil (Müller-Nordhorn et al., 2008; Krasinska et al., 2017). Tomatoes, tomato sauce, and watermelon are important sources of lycopene and may be a surrogate for the Mediterranean diet to some degree (Sesso et al., 2003; Burton-Freeman and Sesso, 2014; Naz et al., 2014).

Dietary lycopene is considered to confer cardiovascular benefits, as e.g., consuming at least 7 servings/week of lycopene-based products significantly decreased cardiovascular risk within seven years in postmenopausal women, free from prior cardiovascular disorders and cancer (Sesso et al., 2003). A negative correlation between serum lycopene concentration and cardiovascular mortality was also found in a follow-up study with a large Japanese cohort (Ito et al., 2006). Many studies about the relationship between lycopene and cardiovascular risk have been conducted and, although, some of the results are inconsistent, overall dietary lycopene intake and high-serum concentration of lycopene, significantly reduced the risk of major cardiovascular events (Cheng et al., 2017; Song et al., 2017). Not all outcomes were positive, as exemplified in the Physicians Health Study, including 499 patients with cardiovascular disorders, not revealing any association of higher plasma lycopene and CVD (Müller et al., 2016). Within the Kuopio Ischaemic Heart Disease Risk Factor (KIHD), no relationship was reported between low serum lycopene and an increased CVD mortality and sudden cardiac death, respectively, in Finnish, middle-aged men (Karppi et al., 2012; Müller et al., 2016).

Vascular health depends on endothelial function, arterial stiffness, and the presence of atherosclerotic plaques. The following section will discuss the different methods to measure vascular health. *Endothelial dysfunction* enables development of the atherosclerotic plaque. The flow mediated dilatation of the brachial artery was traditionally used to assess endothelial function for a long period, but forearm plethysmography and reactive hyperemia—peripheral arterial tonometry have become the gold standards for assessing endothelial vascular function (Kim et al., 2011; Gajendragadkar et al., 2014). While hyperemia—peripheral arterial tonometry is less operator-dependent and non-invasive, while being as reliable as the traditional method, forearm plethysmography additionally provides mechanistic information related to nitric oxide synthesis, is a marker for cardiovascular risk, and can improve risk prediction (Kim et al., 2011; Gajendragadkar et al., 2014).

An increased *arterial stiffness* is one of the first structural and functional changes of the vessel wall and is mainly caused by arteriosclerosis, atherosclerosis and vessel wall calcification (Cavalcante et al., 2011). Arterial stiffness is associated with cardiovascular risk and predicts cardiovascular disorders and mortality (Vlachopoulos et al., 2010; Mozos et al., 2017c). Pulse wave velocity (PWV) and augmentation indices are simple, noninvasive, inexpensive and validated methods used to assess arterial stiffness and as screening methods for the detection of pre-clinical cardiovascular disorders (Vlachopoulos et al., 2010; Mozos et al., 2017a).

*Carotid intima-media thickness* (IMT) assessed non-invasively by B-mode ultrasonography, is a simple, validated and safe method used to measure the extent of subclinical atherosclerosis (Dwyer et al., 2004; Wood and Johnson, 2004; Hosseini et al., 2017; Kim and Youn, 2017). IMT is defined by the measurement of the dimension of the intima and media of the arterial wall, whereas a value >0.9 mm is considered abnormal (Cooney et al., 2015; Piepoli et al., 2016). Although IMT has been considered as a surrogate measure of cardiovascular risk, predicting cardiovascular events, especially myocardial infarction and stroke (O'Leary et al., 1999; Touboul, 2015; Piepoli et al., 2016; Hosseini et al., 2017; Pleskovic et al., 2017), both the American and the European guidelines on cardiovascular disease prevention do not recommend the systematic measurement of IMT to improve risk assessment (Goff et al., 2014; Piepoli et al., 2016). Major concerns of IMT include lack of standardization, its high variability and low intra-individual reproducibility (Piepoli et al., 2016). Additionally, a trained sonographer is required to limit variability of the results due to the patient or medical equipment (Touboul, 2015). There are also methods available that can better predict cardiovascular events than IMT, most prominently MRI (magnetic resonance imaging) that can be used to monitor atherosclerotic plaques. Several methods are stronger predictor of myocardial infarction than IMT, such as monitoring atherosclerotic plaques and exploring the carotid wall by MRI (because the adventitia is also included in the measurement of the wall thickness; Zhang et al., 2014). However, the resources needed to access wall thickness by MRI are manifold higher than using ultrasonography, but especially for patients with abnormal findings in IMT screenings, a subsequent MRI scan might be useful to provide a more accurate description of the atherosclerotic plaque and the risk of major cardiovascular events (Zhang et al., 2014).

Despite the inevitable influence of aging, vascular changes are at least partially reversible, and dietary changes may improve vascular function. Lycopene has several advantages, such as almost no adverse reactions, its wide availability and low cost (Gao et al., 2016). The present review aims to give a current overview of the mechanisms linking lycopene in the human diet and vascular changes, considering epidemiological data, clinical observational, retrospective, intervention and randomized studies, dietary and biomarker studies, *in vitro* and *in vivo* research and discussing preventive benefits of lycopene intake in context of cardiovascular disease prevention.

## Lycopene

**Figure 1:**
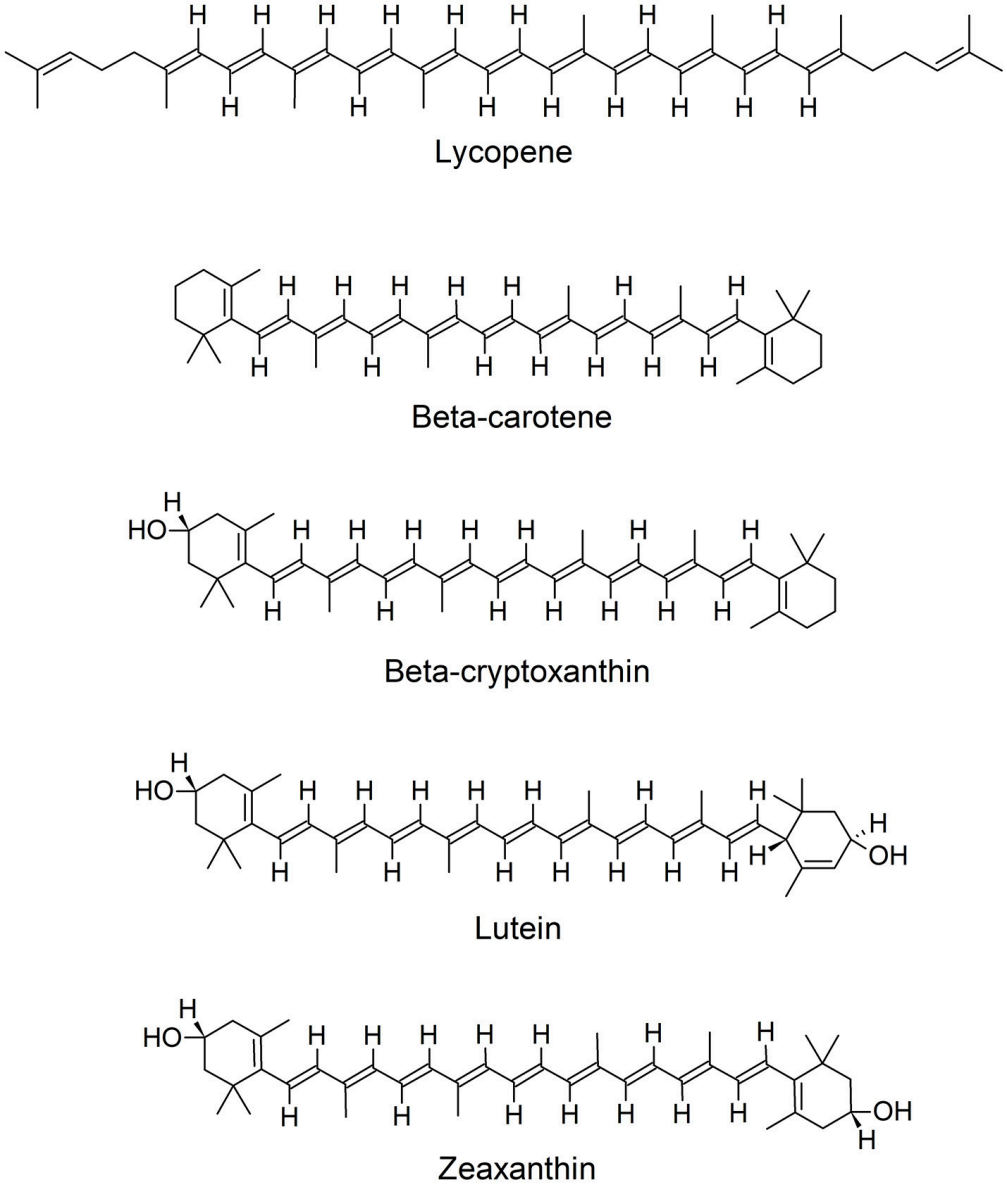
Chemical structures of lycopene and other carotenoids.

Lycopene is a lipophilic, biologically active, unsaturated, acyclic carotenoid, with the chemical formula C_40_H_56_ (Figure 1). In plants it is considered as an important intermediate of carotenoid synthesis, but in human nutrition lycopene plays no role as provitamin A precursor due to the absence of appropriate enzymes. Plant lycopene is usually present as all-trans isomer. Isomerization of the all-trans isomer into the more bioavailable cis-isomer, occurs under acidic conditions (e.g., gastric acid), due to exposure to light and thermal energy. Lycopene can be found in tomatoes, watermelon, papaya, red grapefruits, apricots, and guava, and gives their red color (Kong et al., 2010; Gajendragadkar et al., 2014; Mozos et al., 2017c). Lycopene content increases during different stages of ripening of fruits, e.g., for tomatoes there is a steady increase in lycopene content from the breaker to the red stage (Saini et al., 2017). Watermelon pulp can also be used for lycopene extraction (Oberoi and Soqi, 2017) and is a rich source of cis-isomeric lycopene, abundant in higher concentrations than in tomatoes (Naz et al., 2014). Interestingly also the fungal plant pathogen *Blakeslea trispora* has been recognized as a commercial source to produce lycopene (Mantzouridou and Tsimidou, 2008). Several foods high in lycopene content are classified as functional foods (Naz et al., 2014). Tomato juice, paste, puree, ketchup, sauce or soup represent lycopene sources with improved bioavailability due to thermal treatment, but also because processing releases lycopene from the fibrous cell structure matrix (Basu and Imrhan, 2007; Thies et al., 2012; Burton-Freeman and Sesso, 2014).

Not only lycopene intake counts, but also its serum concentration may influence cardiovascular risk. Low serum and adipose tissue lycopene levels were correlated with early atherosclerosis, and major acute coronary and cerebrovascular events, and were found to be more reliable in risk assessment than the daily intake of lycopene (Agarwal and Rao, 2000; Rissanen et al., 2001; Kim et al., 2011). Oxidative stress and inflammation are responsible for a reduced level of antioxidants (Kim et al., 2010). Smoking is a potent oxidative stressor, able to impair arterial elasticity and endothelial function (Kim et al., 2010; Mozos et al., 2017b), and lycopene was the only major serum carotenoid able to reduce the atherosclerotic risk in current and former smokers according to the Rotterdam Study (Klipstein-Grobusch et al., 2000).

## Cardiovascular protective mechanisms

**Figure 2:**
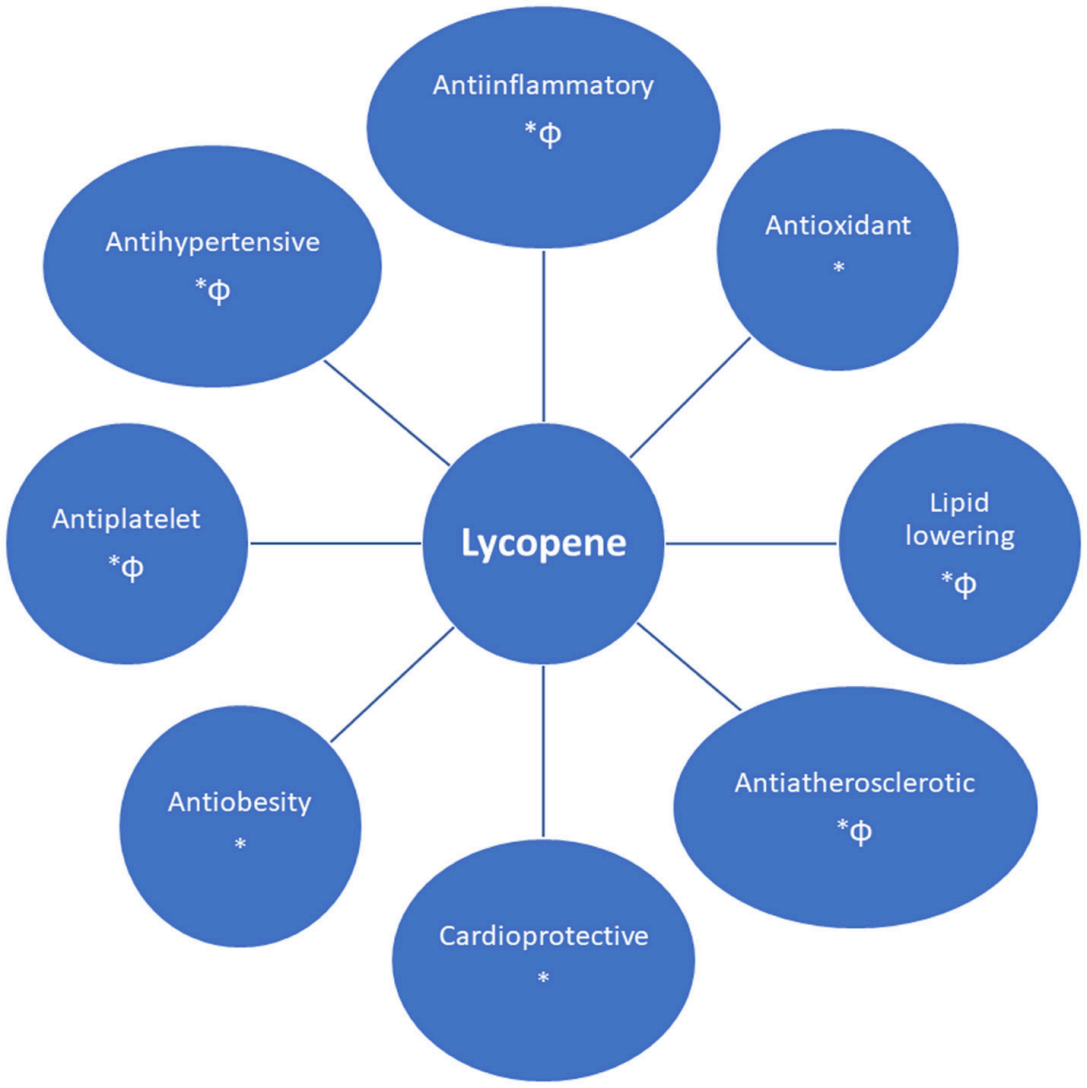
Cardiovascular benefits of lycopene [supported by *in vivo* (^*^) and/or *in vitro* (Φ) findings].

Lycopene has several cardiovascular beneficial effects, such as an antioxidative, antiinflammatory, anti-atherogenic, cardioprotective, and antiplatelet effect, improving endothelial function (nitric oxide bioavailability and blood flow), the metabolic profile (by impairing cholesterol synthesis) and blood pressure control (Figure 2) (Klipstein-Grobusch et al., 2000; Heber and Lu, 2002; Ahuja et al., 2006; Basu and Imrhan, 2007; Verghese et al., 2008; Kim et al., 2010; Kong et al., 2010; Riccioni et al., 2010; Ried and Fakler, 2011; Böhm, 2012; Wolak and Paran, 2013; Gajendragadkar et al., 2014; Naz et al., 2014; Müller et al., 2016; Cheng et al., 2017; Milani et al., 2017).

### Antioxidative and protective endothelial effects

Lycopene is considered an effective singlet oxygen quencher in the carotenoids group (Kong et al., 2010; Viuda-Martos et al., 2014). It is a much more potent *antioxidant* than alpha-tocopherol (10 × more potent) or beta-carotene (twice as potent) (Kim et al., 2010; Kong et al., 2010). Lycopene modulates also the production of antioxidant enzymes, such as superoxide dismutase and catalase (Böhm, 2012; Pereira et al., 2017). Lycopene can also scavenge peroxynitrite, resulting oxidized lycopene products (Pisoschi and Pop, 2015).

Oxidative stress causes endothelial dysfunction due to uncoupling of the nitric oxide synthase and oxidative injury of the endothelial cells (Mozos and Luca, 2017). Both are associated with inflammation. By reducing oxidative stress and reactive oxygen species, lycopene increases the bioavailability of nitric oxide (NO), improves endothelium-dependent vasodilation and reduces protein, lipids, DNA, and mitochondrial damage (Hollman et al., 2011; Naz et al., 2014; Nakamura et al., 2017; Abdel-Daim et al., 2018).

Endothelial NO enables vasodilation, inhibits platelet functions, and adhesion and transmigration of white blood cells, and reduces smooth muscle cell proliferation (Opatrilova et al., 2017). Watermelon supplementation, due to L-citrulline content, increases plasma L-arginine, enabling NO production (Figueroa et al., 2017), because NO is synthesized from L-arginine by NO synthase in virtually all cell types (Jobgen et al., 2006). Lycopene supplementation improved endothelial mediated vasodilation in cardiovascular disease patients, but not in healthy controls (Gajendragadkar et al., 2014), suggesting the importance of lycopene in secondary cardiovascular prevention (Costa-Rodrigues et al., 2018).

In summary, lycopene scavenges both reactive oxygen and nitrogen species, increases the production of antioxidant enzymes and protects the endothelial cells from oxidative damage.

### Anti-inflammatory effect

Inflammation is related to atherosclerosis, arterial stiffness, and major cardiovascular events. The *anti-inflammatory* role of lycopene was demonstrated by several studies (Hung et al., 2008; Kim et al., 2010; Xu et al., 2012; He et al., 2016). Hung et al. revealed that lycopene can inhibit TNF-alpha induced NF-kappa B activation, expression of intracellular adhesion molecule-1 (ICAM-1), and interaction between monocytes and endothelial cells, which might explain the cardiovascular benefits of lycopene (Hung et al., 2008). In a different study with Korean women, lycopene levels were found to correlate with cytokines, but no correlation with acute phase reactants was found, probably due to lycopene's inhibitory effect on the formation of oxidized LDL (Kim et al., 2010). Xu et al. found an inverse association of lycopene with vascular cell adhesion protein 1 (VCAM-1), which enable adhesion of monocytes to the endothelial cells, but could not verify any association between serum lycopene concentration and atherosclerosis in their study as suggested by earlier studies (Xu et al., 2012). In the same context, Gianetti et al. reported no significant correlations between plasma lycopene and soluble adhesion molecules (Gianetti et al., 2002). Lycopene obtained from red guava exerts several anti-inflammatory effects besides modulation of inflammatory mediators, such as inhibition of leukocyte mobilization, stabilization of mast cells, and inhibition of genes which expression is involved in inflammation (Vasconcelos et al., 2017).

Lycopene can also reduce the secretion of metalloproteinases by macrophages and inhibit T lymphocyte activation (Thies et al., 2017). Recently, lycopene was found as an effective antiglycation agent, able to reduce the synthesis of advanced glycation end-products (AGE), downregulating the expression of their receptors (RAGE), which further contributes to vessel protection (Tabrez et al., 2015; Thies et al., 2017).

Tomato products reduced oxidative stress related to postprandial lipemia and the associated inflammatory reaction in a study including normal weight participants (Burton-Freeman et al., 2012). He et al. reported the benefits of lycopene in preventing transplant vasculopathy, demonstrating that intimal hyperplasia and smooth muscle cell proliferation were reduced by the administration of lycopene and the infiltration of inflammatory cells in allograft vessels was reduced in an animal model (He et al., 2016). Lycopene can ameliorate allograft atherosclerosis via downregulating Rho-associated kinases and regulating the expression of key factors through NO/cGMP pathways (He et al., 2016). On the other hand, the benefits of the tomato-rich diet were not directly related to the anti-inflammatory effect according to a randomized study including 103 apparently healthy volunteers, after 300 g tomatoes daily for 1 month or placebo (Blum et al., 2007).

Watermelon was shown to reduce levels of inflammation by downregulation of the proinflammatory mediator cyclooxygenase 2 (COX-2), impairing prostaglandin E2 and I2 production, which reduces the progression of cardiovascular disorders (Sellers et al., 2010; Hong et al., 2015). Watermelon powder supplementation exerts an anti-inflammatory effect similar to COX-2 inhibitors or conventional non-steroidal anti-inflammatory drugs (Hong et al., 2015).

High mobility group box 1 (HMGB1), a non-histone DNA binding protein, produced by necrotic and immune cells, exposed to pro-inflammatory signals, has an important pro-inflammatory effect by attracting and activating inflammatory cells and mediators and binding to RAGE and toll-like receptors, related to fatal outcomes (Lee et al., 2012). Lee et al. demonstrated that lycopene inhibits adhesion molecules expression, which impair HMGB1—induced monocyte adhesion and transmigration (Lee et al., 2012). Lycopene has been also shown to inhibit lipopolysaccharide-induced HMGB1 release and HMGB1-mediated secretion of TNF-alpha and secretory phospholipase A2 (Lee et al., 2012).

Oxysterols, the result of cholesterol auto-oxidation, accumulate in the subendothelial arterial layer, exerting oxidative and pro-inflammatory roles and favoring the atherosclerotic process (Palozza et al., 2011). Lycopene impairs oxysterol-induced pro-inflammatory cytokines production in human macrophages and oxysterol-induced ROS production, limiting the formation of atherosclerotic plaque (Palozza et al., 2011).

Lycopene exerts a cardioprotective effect against atrazine induced cardiac injury due to its anti-inflammatory effect, by blocking the NF-kappa B pathway and NO production (Li et al., 2017).

**Figure 3:**
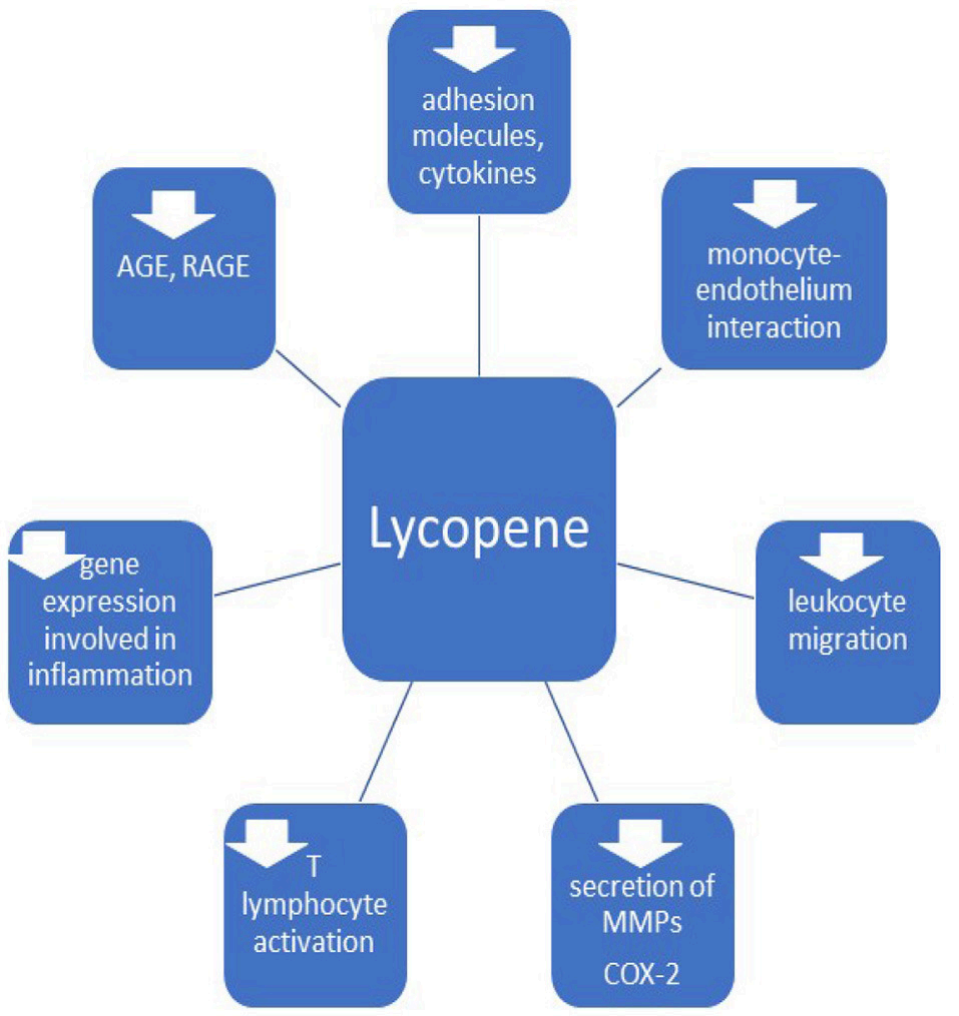
Anti-inflammatory effects of lycopene. MMPs, matrix metalloproteinases; COX-2, cyclooxigenase 2; AGE, advanced glycation end-products; RAGE, receptors of AGE.

Considering the mentioned anti-inflammatory mechanisms (Figure 3), including decrease of adhesion molecules, pro-inflammatory cytokines, inhibition of leukocyte migration and genes involved in inflammation, impaired monocyte-endothelium interaction, T lymphocytes activation and synthesis of AGE and RAGE and downregulation of cyclooxygenase 2, lycopene can be useful in the therapy for vascular inflammatory disorders.

### Modulation of lipids

Lycopene is transported in the circulation by lipoproteins and is actively taken up into adipocytes (McEneny et al., 2013). Lycopene is a *regulator of cholesterol* levels by inhibition of HMG-CoA reductase (like statins) and down-regulation of proprotein convertase subtilisin/kexin type 9 mRNA synthesis. These results suggest that lycopene supplementation could be especially beneficial for patients with statin intolerance (Sultan Alvi et al., 2017). Changes in hepatic gene expression, such as reduced expression in fatty acid synthase, responsible for fatty acid storage, were described after watermelon powder consumption in rats (Hong et al., 2015). Carbonic anhydrase III and adenylate kinase 2 were involved in the lipid-lowering and antioxidant effects of tomatoes (Hsu et al., 2008). On the other hand, NO stimulates fatty acid oxidation and lipolysis in adipose cells (Jobgen et al., 2006; Hong et al., 2015).

Lycopene is not able to increase HDL cholesterol, but was shown to improve the LDL/HDL ratio, HDL functionality and reduced the accumulation of cholesterol in the rabbit aorta, underlining the beneficial effects of lycopene during initial stages of atherosclerosis (Lorenz et al., 2012; Thies et al., 2017). However, a recent meta-analysis found that significant reductions in total and LDL cholesterol were revealed only at doses of, at least, 25 mg lycopene/day in human subjects, and the effects were comparable with low-dose statin medication, without concomitant side effects (Ried and Fakler, 2011; Lorenz et al., 2012). Fecal cholesterol excretion increases with levels of dietary lycopene due to decreased intestinal cholesterol absorption. This can be explained by the insight that increased fecal excretion impairs the enterohepatic circuit of bile acids, thus increasing the conversion of cholesterol to bile acids (Verghese et al., 2008). The lipid lowering properties of lycopene involve also an increased activity of LDL receptors in macrophages (Li et al., 2015; Cheng et al., 2017).

Lycopene can lower synthesis of dysfunctional HDL, modulating HDL functionality toward an antiatherogenic phenotype, with a low serum amyloid A level and beneficial changes of the activity of HDL remodeling enzymes (cholesterol ester transfer protein and lecithin cholesterol acyl transferase) (McEneny et al., 2013). A hypotriglyceridemic effect of tomato juice was seen only in subjects with initial high serum triglyceride levels (Li et al., 2015).

Lycopen can also regulate the hepatic lipid metabolism and counteracts the hepatic steatosis induced by a high-fat diet, due to sirtuins (SIRT1) induction and activation, being able to suppresses lipogenesis, to stimulate lipid catabolism in the liver and skeletal muscles and lipid mobilization in the white adipose tissue (Lomb et al., 2010; Li et al., 2015). Another mechanism able to ameliorate liver steatosis, by lycopene, was described in mice and is related to decrease of fatty acid binding protein 7 due to binding to microRNA-21 (Ahn et al., 2012; Li et al., 2015).

In summary, lycopene has lipid lowering properties, reducing the total and LDL cholesterol, triglyceride level, LDL oxidation, and synthesis of dysfunctional HDL.

### Anti-aggregative effect

Platelets are involved in the pathogenesis of the atherosclerotic plaque, development of acute thrombotic events and restenosis after endovascular procedures (Krasinska et al., 2017; O'Kennedy et al., 2017). Another cardiovascular beneficial effect of lycopene, protecting against myocardial infarction and stroke, is its *antiplatelet* activity, which is concentration dependent, and was demonstrated *in vivo* and *in vitro* (Sawardekar et al., 2016). Several mechanisms were considered in explaining the reversible antiplatelet effect of lycopene, such as the interaction with thromboxane, thrombin, collagen, von Willebrand factor, P-selectin and inflammatory mediators, the influence on calcium and cyclic guanosine monophosphate signaling and ADP-mediated aggregation (Sawardekar et al., 2016; Krasinska et al., 2017). It was noticed that lycopene can potentiate the antiplatelet effect of aspirin, which requires low lycopene diet in patients on secondary prophylaxis with aspirin due to the potential bleeding risk (Sawardekar et al., 2016). On the other hand, in high cardiovascular risk, aspirin (ASP) resistant patients, or those with ASP contraindications, high risk of complications after antiplatelet therapy or hyperactive platelets (obese, sedentary, hypertensive, diabetic, aging patients, and smokers), lycopene could have an important contribution in cardiovascular prophylaxis (Sawardekar et al., 2016; Krasinska et al., 2017; O'Kennedy et al., 2017).

### Antihypertensive effect

Lycopene has antihypertensive effects due to inhibition of the angiotensin converting enzyme (ACE) and due to its antioxidant effect, reducing oxidative stress induced by angiotensin-II and indirectly enhancing production of nitric oxide in the endothelium (Li and Xu, 2013; Belovic et al., 2016; Khan et al., 2016; Han and Liu, 2017). A study including 8,556 adult overweight and obese participants demonstrated association of lycopene and lycopene/uric acid ratio with lower prevalence of hypertension (Han and Liu, 2017). Paran et al. reported a decrease in both systolic and diastolic blood pressure in 54 patients with moderate hypertension, treated with ACE inhibitors or calcium channel blockers, after 6 weeks of tomato extract supplementation, suggesting a cause-effect relationship (Paran et al., 2009). Li et al. concluded, in a metanalysis, that lycopene supplementation (more than 12 mg/day) might significantly reduce systolic, but not diastolic blood pressure, in prehypertensive or hypertensive patients (Li and Xu, 2013).

Angiotensin II induces, besides direct vasoconstriction and oxidative stress, also vascular smooth muscle cells phenotypic transformation and production of inflammatory cytokines (Ren et al., 2017), and lycopene might impair the mentioned pathways, as well.

### Anti-atherosclerotic mechanisms

**Figure 4:**
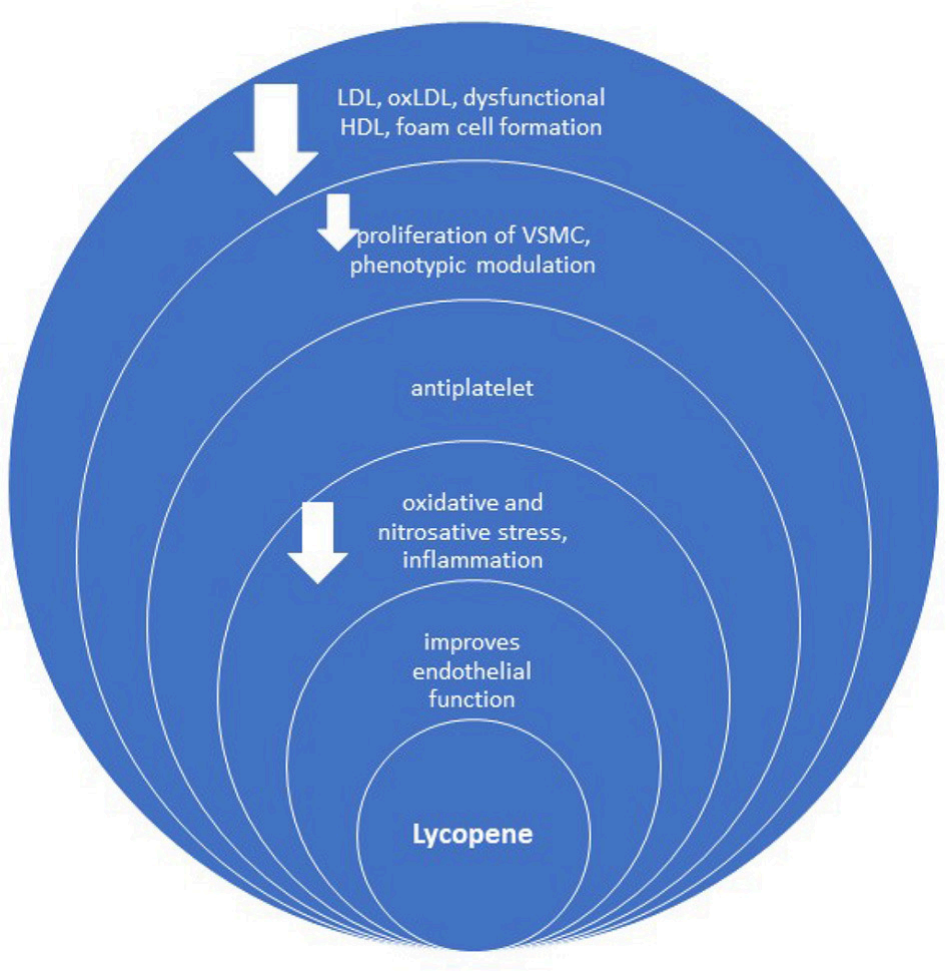
Anti-atherosclerotic effect of lycopene. oxLDL, oxidized LDL; VSMC, vascular smooth muscle cells.

Besides improving endothelial function, oxidative stress (preventing oxidation of LDL) and metabolic profile, the anti-inflammatory and antiplatelet effect, lycopene has several other anti-atherosclerotic contributions (Figure 4), such as inhibition of vascular smooth muscle cell (VSMC) proliferation and foam cell formation (Napolitano et al., 2007; Wang et al., 2014). In general, not all studies confirm the relationship between lycopene and early atherosclerosis (Kim et al., 2010).

Contractile VSMC change to a proliferative and migratory phenotype during the atherosclerotic process, enabling migration of VSMC into the intima and production of the extracellular matrix of the plaque (Karagiannis et al., 2013). Such changes in phenotype are called “phenotypic modulation” (Manabe and Nagai, 2003) and play an important role in vascular remodeling, not only due to atherosclerosis, but also in hypertension and diabetic macroangiopathy (Ren et al., 2017). Lycopene can suppress VSMCs proliferation, due to inhibiting G1 phase cells entry into the S phase of the cell cycle, related to its antioxidative effect (Chen et al., 2010), and not due to the inhibition of matrix metalloproteinases (Lo et al., 2007). Minimally-oxidized LDL can induce phenotypic modulation of VSMC (Karagiannis et al., 2013), and synthesis of oxidized LDL may be impaired by lycopene (Kim et al., 2010). Lycopene may block VSMC migration and proliferation also by direct binding to platelet-derived growth factor (PDGF) and inhibiting PDGF-signaling (Lo et al., 2007), or due to it antioxidant effect, considering that reactive oxygen species accelerate the switch from the contractile to the synthetic phenotype (Sung et al., 2005).

Lycopene has also barrier integrity activity in the endothelial membrane, by blocking the activation of CD14 and Toll like receptor-4 expression (Bae and Bae, 2011) and impairs the apoptosis of endothelial cells *in vitro*, by disrupting the upregulation of p53 and caspase 3 mRNA (Tang et al., 2009).

### Cardioprotective effects

Recent studies emphasized the cardioprotective effects of lycopene. An experimental study including rats with isoproterenol induced myocardial infarction, demonstrated improved ECG findings (shorter QT and RR intervals and QRS complexes, reduced ST segment elevation) if lycopene was previously administrated, related to its cell membrane stabilizing and antioxidant properties (Aman et al., 2012). Supplementation of lycopene in the same study also prevented alteration in hemodynamic parameters (systolic, diastolic, and mean blood pressure), biochemical and inflammatory markers, apoptotic changes, and reduced the size of myocardial infarction due to the antioxidant and anti-inflammatory properties of lycopene (Aman et al., 2012; Wong et al., 2017).

Tomato and lycopene supplementation attenuated cardiac-remodeling and improved diastolic dysfunction after myocardial infarction according to a study including male Wistar rats, enabled by impairing type I and type III collagen content in the left ventricle, reduced cardiomyocyte death, expression of miRNAs and by the anti-inflammatory effect and reduction of oxidative stress (Pereira et al., 2017). Wang et al. administered lycopene in infarcted rats for 28 days, revealing an increased ejection fraction compared to controls, associated with decreased collagen fraction in the peri-infarct zone, due to inhibition of p38 mitogen-activated protein kinase and matrix metalloproteinase 9 expression (Wang et al., 2014). Lycopene effect on interstitial collagen accumulation was not confirmed by Anjos Ferreira et al., in a study including male Wistar rats and testing the effect of lycopene on doxorubicin-induced cardiotoxicity (Anjos Ferreira et al., 2007). The anti-inflammatory effect of lycopene has also a contribution in reversing ventricular remodeling, by inhibiting the nuclear factor-κB signaling pathway (He et al., 2015).

Lycopene might also serve as a cardioprotective agent against several drugs. The cardioprotective effect of lycopene was demonstrated in the case of tulathromycin, a macrolide antibiotic, and diclofenac sodium, a non-steroidal anti-inflammatory drug, by Abdel-Daim et al., in a study including Swiss albino mice, and was attributed to its antioxidant activity (Abdel-Daim et al., 2018). Lycopene can also protect against the cardiotoxicity induced by doxorubicin (Karimi et al., 2005; Anjos Ferreira et al., 2007; Abushouk et al., 2017), isoproterenol (Aman et al., 2012; Mohamadin et al., 2012), and atrazine (Li et al., 2017).

Concluding, lycopene supplementation is beneficial for early and late prognosis in myocardial infarction. It may reduce myocardial infarction size and related electrocardiographic, hemodynamic, and biochemical changes (Aman et al., 2012). Lycopene also reverses ventricular extracellular matrix remodeling after an acute coronary event, by inhibiting myocardial fibrosis and preventing apoptosis and necrosis of cardiomyocytes, its anti-inflammatory effect and the ability to increase left ventricular function, preventing development of heart failure and increasing survival rates (Aman et al., 2012; Wang et al., 2014; He et al., 2015). Considering difficulties and high cost of early revascularization and significant side effects of cardiovascular drugs, lycopene could represent a safe and effective option in controlling post infarction ventricular remodeling (Wang et al., 2014).

### Lycopene and endoplasmic reticulum stress (ERS)

The endoplasmic reticulum has several functions related to intracellular calcium storage, lipid and protein synthesis, and modification (Schönthal, 2012). Disturbance of those functions causes “endoplasmic reticulum stress” (ERS) involved in myocardial ischemia/reperfusion injury (Gao et al., 2016). The cell reacts to ERS by initiating an “unfolded protein response,” including several mechanisms, enabling cellular adaptation and survival or, in severe cases, apoptosis (Schönthal, 2012).

Lycopene protects the cardiomyocytes by relieving ERS and preventing apoptosis, through stimulation of adenosine 5-monophosphate-activated proteinkinase, the transcription factor CHOP (C/EBP homologous protein), p-JNK and Caspase pathways (Xu et al., 2015; Gao et al., 2016). However, high lycopene levels might be toxic to the cardiomyocytes (Gao et al., 2016).

## Preclinical studies

Lycopene supplementation for 4 weeks strongly reduced total and LDL serum cholesterol and the amount of cholesterol in the aorta, but the surface lipid accumulation in the aorta and IMT were not significantly reduced and no impairment of vasoreactivity or increase of pro-oxidant parameters were detected in New Zealand White rabbits after either a standard or a high-cholesterol diet (Lorenz et al., 2012). Although lycopene suppressed cholesterol uptake and intestinal absorption and increased fecal cholesterol excretion in rabbits, it did not decrease the expression of HMG-CoA reductase (Lorenz et al., 2012).

Hu et al. reported anti-atherosclerotic effects for both lycopene and fluvastatin, in the aorta, in rabbits, after a high-fat diet (Hu et al., 2008; He et al., 2009). Verghese et al. also revealed a significant decrease in the atherosclerotic plaque formation with the consumption of lycopene in a study including New Zealand male rabbits, receiving a high cholesterol diet. Further observed effects include an improved serum lipid profile as well as reduction in total cholesterol, LDL and triglycerides (Verghese et al., 2008). Hsu reported, besides reduction in total and LDL cholesterol and plasma malondialdehyde (MDA) levels, also an increase in HDL cholesterol in hamsters fed a 9% tomato paste containing 0.2% cholesterol, after 8 weeks (Hsu et al., 2008).

Watanabe Heritable Hyperlipidemic rabbits were used in a study by Frederiksen et al. demonstrating that lycopene did not influence cholesterol and triacylglycerol levels, lipoprotein fractions, oxidation of lipids, and aortic atherosclerosis evaluated biochemically and by microscopy (Frederiksen et al., 2007). The lack of response of lycopene in Watanabe Heritable Hyperlipidemic rabbits is, probably, related to their defective LDL receptors (Tanazawa et al., 1980; Lorenz et al., 2012), which would suggest the involvement of these receptors in the cardiovascular benefits of lycopene (Perera and Yen, 2007). Considering the bloodstream transport of lycopene in LDL particles, functional LDL receptors enable cardiovascular benefits of lycopene (Lorenz et al., 2012). Bansal et al. reported a beneficial cardioprotective effect of lycopene, due to the reduction of oxidative stress and myocardial injury, in an experimental model of myocardial ischemia-reperfusion injury in adult male albino Wistar rats (Bansal et al., 2006). Lycopene protects also endothelial progenitor cells, necessary to replace the injured vascular endothelium and for angiogenesis, in a microenvironment of advanced glycation end products (AGEs), which act as damage-causing agents (Zeng et al., 2017). Lycopene improved cell proliferation and regulated protective mechanisms of AGEs-induced autophagy in endothelial progenitor cells from diabetic rats, suggesting that supplementation with this compound might be a new therapeutic option for diabetic vascular complications (Zeng et al., 2017).

## Clinical research

### Endothelial function

Both cross-sectional and supplementation studies emphasized the benefits of tomato products on vascular function, mainly due to the antioxidative effects of lycopene (Kim et al., 2010; Thies et al., 2012; Xaplanteris et al., 2012; Gajendragadkar et al., 2014). However, several articles failed to show improvement of endothelial function after tomato consumption (Stangl et al., 2011; Table 1).

**Table 1:** Effects of lycopene on endothelial function and arterial stiffness.

**Number of participants**	**Methodology**	**Results, conclusions**	**References**
36 statin treated cardiovascular patients (mean age: 67–68 years) and 36 healthy volunteers (mean age: 61–68 years)	Double-blind trial: 7 mg lycopene or placebo daily for 2 months.	Lycopene supplementation improved endothelial function in patients with cardiovascular disorders on optimal secondary prevention, but not in healthy persons.	Gajendragadkar et al., 2014
225 overweight volunteers, aged 40–65 years	Participants were randomly assigned into 1 of 3 dietary intervention groups: control diet (low tomato content), a high-tomato-content diet, or a control diet with addition of lycopene capsules (10 mg/d) for 12 wk. Collected blood samples were tested for carotenoid and lipid profiles and inflammatory markers. Arterial stiffness and dietary intake were also monitored.	A relatively high daily consumption of tomato-based products (32–50 mg lycopene/day) or lycopene supplements (10 mg/day) was ineffective in reducing conventional cardiovascular risk markers, inflammatory markers, markers of insulin resistance and sensitivity, lipid profile and arterial stiffness in moderately overweight, healthy, middle-aged individuals.	Thies et al., 2012
25 study participants, mean age: 27 ± 8 years	Randomized, intervention model, crossover assignment. The participants consumed high-fat meals containing processed tomato products or non-tomato alternative.	Tomato products attenuate postprandial lipemia-induced oxidative stress and inflammatory response, with a modest improved flow-mediated dilatation (FMD).	Burton-Freeman et al., 2012
19 volunteers, 39 ± 13 years	Randomized, single-blind, crossover assignment.	Daily tomato paste consumption exerts a beneficial midterm effect on endothelial function.	Xaplanteris et al., 2012
299 Korean men	Subgrouped according to the number of metabolic syndrome risk factors; brachial-ankle pulse wave velocity (PWV), oxidative stress and antioxidants (including lycopene) were measured.	An inverse correlation was found between PWV and serum lycopene, considering blood pressure, insulin resistance and oxidative stress.	Yeo et al., 2011
19 healthy non-smoking postmenopausal women	Administration of 70 g tomato puree. Endothelial-dependent FMD and endothelial-independent nitro-mediated dilation of the brachial artery were measured with high-resolution ultrasound.	Acute and long-term (7 d) intake of tomato products, despite a significant increase in plasma lycopene had no effect on endothelial function.	Stangl et al., 2011
126 healthy men	Administration of placebo/6 mg/15 mg lycopene daily for 8 weeks. Endothelial function was assessed by reactive hyperemia peripheral arterial tonometry. Plasma superoxide dismutase was used to assess oxidative stress.	An inverse correlation between serum lycopene levels and arterial stiffness was found. An increased serum lycopene decreases oxidative stress, which might influence endothelial function.	Kim et al., 2011
264 healthy women, 31–75 years	The relationship between serum lycopene and brachial-ankle pulse wave velocity was assessed.	An independent, inverse relationship between circulating lycopene and brachial PWV was observed.	Kim et al., 2010

Gajendragadkar et al. concluded that lycopene supplementation can improve endothelial function in patients with cardiovascular disorders, but not in age-matched healthy volunteers (Gajendragadkar et al., 2014). Forearm responses to intraarterial infusions of acetylcholine were assessed using venous plethysmography, which resulted in an improvement by 53% of endothelium-dependent vasodilatation (EDV) in patients with cardiovascular disorders post-lycopene (Gajendragadkar et al., 2014). Endothelial function was improved regardless of traditional risk factors or inflammatory markers, and, even a modest increase in serum lycopene, further impaired endothelial function in atherosclerotic patients (Gajendragadkar et al., 2014). One conclusion of the mentioned study was that lycopene affects especially smaller vessels, such as resistance arteries, rather than larger vessels, as measured arterial stiffness remained unaltered in all study participants (Gajendragadkar et al., 2014).

### Pulse wave velocity (PWV)

Kim et al. reported an independent, inverse association between circulating lycopene and brachial pulse wave velocity in 264 healthy women, regardless of age, body mass index, smoking and drinking habits, menopause, blood pressure, beta-carotene, alpha-tocopherol, markers of oxidative stress, and inflammation (Kim et al., 2010; Table 1). Reduced oxidative LDL changes may have an important contribution to arterial stiffness reduction due to lycopene (Kim et al., 2010). Another study, including 126 healthy men revealed the benefits of lycopene on oxidative stress and endothelial dysfunction, especially in subjects with an impaired endothelial function (Kim et al., 2011). After 15 mg/day lycopene supplementation, for 8 weeks, Kim et al. reported a decrease of systolic blood pressure and high sensitivity C reactive protein (Kim et al., 2011).

The main biological mechanism by which lycopene reduces the risk and mortality of the *metabolic syndrome*, include the antioxidant, anti-inflammatory and antiobesity effects, the ability to improve endothelial function, glycemic control, insulin sensitivity and lipid profile (Tsitsimpikou et al., 2014; Li et al., 2015; Han et al., 2016a,b). An inverse relationship was found by Yeo et al. between lycopene level and brachial-ankle pulse wave velocity (Yeo et al., 2011). PWV was significantly higher in patients with metabolic syndrome, with lower than median serum lycopene values (≤ 0.0294 mmol/l) compared to patients without metabolic syndrome; no statistically significant differences between the 2 groups were found when lycopene levels were high (Fantin et al., 2010; Yeo et al., 2011). Higher serum carotenoid levels were associated not just with a lower prevalence of the metabolic syndrome, but also with fewer abnormal metabolic syndrome components. A significant association between lycopene and the metabolic syndrome was described only for normal-weight and overweight participants, but not in obese patients, according to a study enrolling 13,196 subjects, probably related to an increased oxidative stress and decreased antioxidant ability, due to sequestration of lycopene in the adipose tissue and more important inflammation in obese (Han et al., 2016b). On the other hand, daily tomato juice intake reduced waist circumference, cholesterol, and monocyte chemotactic protein-1 (inflammatory adipokine) and increased adiponectin (anti-inflammatory adipokine) levels in 30 young, healthy Taiwanese females (Li et al., 2015). Lycopene was shown to impair pro-inflammatory cytokine production, such as IL-6, IL-1b, and TNF-α, preventing insulin resistance (Gouranton et al., 2011).

L-arginine or L-citrulline supplementation modulate the arginine-NO pathway, enabled by multiple cyclic guanosine-3′,5′-monophosphate-dependent pathways, with important prophylactic and therapeutic contributions in the metabolic syndrome (Jobgen et al., 2006).

A large study, including 225 middle-aged, overweight volunteers reported no changes of conventional cardiovascular risk factors, inflammatory tests, insulin resistance and sensitivity, lipid profile, oxidized LDL, von Willebrand factor, and arterial stiffness after high daily intake of lycopene, despite good compliance (Thies et al., 2012). One week of lycopene supplementation increased plasma lycopene, but not biomarkers of vascular oxidative stress and inflammation, or biomarkers of nitric oxide (plasma nitrate/nitrite) in healthy, active subjects, suggesting that the participants already possessed a robust antioxidant capacity and lycopene provided no additional benefit (Denniss et al., 2008).

### Intima-media thickness (IMT)

Several studies reported an association between serum lycopene levels and intima-media thickness (Gianetti et al., 2002; Riccioni et al., 2009, 2011; Karppi et al., 2013; Zou et al., 2014), while other authors reported no association (Dwyer et al., 2004; Table 2). Zou et al. revealed a decrease in carotid artery intima-media thickness (IMT) after 12 months of lutein and lycopene supplementation (20 mg each) in 144 Chinese patients with subclinical atherosclerosis, demonstrating more effective results after the intake of both lutein and lycopene compared to lutein alone (Zou et al., 2014).

**Table 2:** Lycopene and intima-media thickness (IMT).

**Number of participants**	**Methodology**	**Results, conclusions**	**References**
144 subjects, aged 45–68 years, with subclinical atherosclerosis	20 mg lutein (*n* = 48), 20 mg lutein + 20 mg lycopene (*n* = 48) or placebo (*n* = 48) were administrated for 12 months; carotid artery intima-media thickness (IMT) was measured using Doppler ultrasonography.	Lutein and lycopene supplementation significantly increased the serum concentration of lutein and lycopene with a decrease in carotid artery IMT.	Zou et al., 2014
840 middle-aged men from Eastern Finland	Ultrasonography of the common carotid arteries, serum levels of carotenoids	7-year change in maximum intima media thickness was inversely associated with serum levels of lycopene, alpha and beta-carotene, respectively. Elevated serum levels of carotenoids may have anti-atherosclerotic effect.	Karppi et al., 2013
1,212 elderly men from Eastern Finland	B-mode ultrasound (IMT of the common carotid artery); plasma levels of carotenoids	High plasma concentrations of lycopene, alpha-carotene and beta-cryptoxanthin are related to decreased carotid atherosclerosis in elderly patients.	Karppi et al., 2011
120 subjects without history of symptomatic carotid artery disease	Ultrasonic measurement of common carotid artery IMT, serum profile analysis of cholesterol (total and LDL), triglycerides and lycopene.	Carotid atherosclerosis was associated with lower plasma lycopene levels.	Riccioni et al., 2011
640 participants with asymptomatic carotid atherosclerosis	Carotid ultrasound investigation was performed; medical history and laboratory data were collected.	Participants with IMT ≥ 0.8 mm had significantly lower concentrations of vitamin A and E, lycopene, and beta-carotene compared to participants with no evidence of carotid atherosclerosis.	Riccioni et al., 2011
573 middle-aged women and men from an occupational cohort	Ultrasound examination of the common carotid arteries was performed, lipid profile and risk factors were assessed at baseline and 18-month follow-up. Plasma antioxidants were determined at baseline.	18-month change in IMT was inversely related to serum levels of some measured carotenoids, not including lycopene, regardless of cardiac risk factors and high-sensitivity C-reactive protein.	Dwyer et al., 2004
11 healthy controls, 11 patients with uncomplicated hypertension, 11 with essential hypertension and peripheral vascular disease	Patients were matched for age, sex, smoking habit and body mass index; IMT, adhesion molecules, LDL and antioxidants (including lycopene) were measured.	A statistically significant correlation was found between lycopene and IMT, independent of LDL, creatinine clearance, and plasma insulin. No significant correlation was found between lycopene and soluble adhesion molecules	Gianetti et al., 2002
1,111 subjects, aged 27–77 years	Dietary vitamin intake, fasting plasma levels of vitamins (A, C, and E), lycopene, alpha and beta-carotene, bilateral	There was an inverse association between carotid artery IMT and plasma lycopene in women, but not in men.	McQuillan et al., 2001

High serum levels of lycopene, alpha and beta-carotene were associated with a slow IMT progression during 7 years in a study including 840 middle-aged men from Eastern Finland (Karppi et al., 2013). The association between lycopene level and IMT was mentioned in the scientific literature also for elderly Finish subjects (Karppi et al., 2011).

Higher carotenoids levels (lutein, zeaxanthin, and beta-cryptoxanthin; Figure 1) were correlated with reduced IMT progression over 18 months, in a study with 573 middle-aged participants, free of cardiovascular symptoms at baseline. Beta-carotene and lycopene levels were not significantly associated with IMT progression (Dwyer et al., 2004).

An inverse correlation was found in women between lycopene levels and IMT, independent of conventional risk factors, in a large study including 1,111 subjects (McQuillan et al., 2001).

## Why conflicting results?

Several studies revealed the anti-atherosclerotic effect of lycopene (McQuillan et al., 2001; Gianetti et al., 2002; Hu et al., 2008; Verghese et al., 2008; Riccioni et al., 2009; Kim et al., 2010; Gajendragadkar et al., 2014), but there are also studies available that report conflicting results related to the vascular effects of lycopene (McQuillan et al., 2001; Dwyer et al., 2004; Stangl et al., 2011; Thies et al., 2012). The possible reasons for this obvious discrepancy are manifold and include methodological differences in the study designs, such as different lycopene sources, the use of food-frequency questionnaires, different intervention times, the methodology used to assess vascular function, measurement of blood, adipose or dietary lycopene. Besides those, the use of unstandardized amounts of tomato food products, different modes of delivery, misclassification of overall tomato intake, combination of lycopene with other antioxidants, different processing procedures or eating behavior influenced by cultural and temporal patterns among different individuals, may influence the results (Sesso et al., 2003; Kong et al., 2010; Thies et al., 2012; Gajendragadkar et al., 2014). Other carotenoids extracted from tomatoes could be also partially responsible for the effects attributed to lycopene (Rao, 2002). This is underlined by a study that could not find beneficial effects for lycopene supplementation alone, but beneficial effects upon supplementation with tomato-based products (Sesso et al., 2003). Some studies did not consider dietary intake at all (Yeo et al., 2011). The interaction flavanone metabolites—lycopene is difficult to assess, considering the rapid metabolization of the mentioned metabolites (Habauzit et al., 2015). Duration of treatment, dose and bioavailability of lycopene, and vascular endpoint were also different in the studies published on this topic and might have influenced obtained results. Several factors influence the bioavailability of lycopene, such as season, the processing of tomatoes, their origin, dimensions, shape, and the way they are consumed (Gajendragadkar et al., 2014; Gammone et al., 2015). Absorption of lycopene may be reduced by diets rich in fibers and in elderly people (Kong et al., 2010) and is increased in the presence of oil.

The isomerization of lycopene is another source of variability. Fresh tomatoes contain lycopene in all-trans form (Shi and Le Maguer, 2000). Several factors, including high temperatures, light, oxygen, acids, and metal ions enable isomerization of lycopene (Kong et al., 2010). Lycopene degradation occurs during thermal processing, mainly isomerization of all- trans to cis forms and oxidation (Shi and Le Maguer, 2000). Dehydrated and powdered tomatoes have poor lycopene stability, depending of storage in a hermetically sealed atmosphere, and a significant increase of cis-isomers, giving the highest bioavailability of lycopene and higher ability to be incorporated in lipoproteins (Shi and Le Maguer, 2000; Kong et al., 2010). Uptake of cis lycopene is significantly higher than all trans-isomers (Kong et al., 2010).

Lycopene is very bioavailable in the presence of oil, especially in monounsaturated oils, other dietary fats and processed tomato products (Shi and Le Maguer, 2000; Basu and Imrhan, 2007; Kong et al., 2010; Gajendragadkar et al., 2014). Lycopene can increase the antioxidant properties of vitamin C, E, polyphenols and beta-carotene in a synergistic way (Kong et al., 2010; Karppi et al., 2013). Supplementation with tomatoes, containing lycopene (red tomatoes) or not (yellow tomatoes), showed a better antioxidant effect than lycopene alone, probably due to the synergistic effects of naturally occurring secondary metabolites in tomatoes (Basu and Imrhan, 2007; Gitenay et al., 2007). Generally, supplementation with whole fruits is often more beneficial than supplementing single food constituents: Watermelons contain, besides lycopene, hundreds of different compounds, including L-citrulline and ascorbic acid, both of which improve the L-arginine/NO pathway, endothelial function, aortic systolic blood pressure, reduce arterial stiffness, and improve glycemic control, thus acting synergistic with lycopene (Wu et al., 2007; Figueroa et al., 2013, 2017). Grapefruits also include in their composition not just lycopene but also flavonoids, with several benefits, such as the anti-inflammatory and anti-atherogenic effect, improving vascular reactivity, reducing insulin resistance, decreasing arterial stiffness, LDL cholesterol, and blood pressure (Habauzit et al., 2015). These synergistic effects hamper assessment of quantitative and qualitative effects of lycopene as a dietary factor.

Several studies included healthy participants or subjects with different disorders and cardiovascular risk factors (Kong et al., 2010; Thies et al., 2012; Gajendragadkar et al., 2014). Enrolling volunteers with established elevated risk markers for cardiovascular disorders may increase the probability of detecting changes, especially in short time studies (Thies et al., 2012). Also, several other uncontrolled or unidentified lifestyle factors or dietary constituents associated with cardiovascular disorders, may provide alternative explanations for the different study results (Sesso et al., 2003). Genetic factors remain unconsidered at all in all of the reviewed publications, although they are reported to strongly influence circulating concentrations of lycopene in different ethnicities (Zubair et al., 2015). Furthermore, plasma, adipose, and dietary carotenoids are not sufficiently correlated to be interchangeably (Sesso et al., 2003).

## Future research directions

Most of the studies considered only tomatoes and tomato products as lycopene source. It will be the aim of future human intervention studies to include other lycopene containing fruits such as watermelon, papaya, red grapefruits, and guava, and consider synergistic effects with other components and their importance in primary and secondary cardiovascular prophylaxis.

Benefits of lycopene should be especially considered in patients with high cardiovascular risk, statin intolerance, borderline hypertension, aspirin resistance, hyperactive platelets, vascular inflammatory diseases, metabolic syndrome and coronary heart disease, and its inclusion in combination therapies for the mentioned disorders, should be approached. Further mechanistic research is needed to identify new targets for prevention and complementary treatment of cardiovascular disorders.

## Conclusions

The present review supports the importance of lycopene in improving vascular function and in the primary and secondary prevention of cardiovascular disorders. The demonstrated effects of lycopene in view of cardiovascular health comprise its general antioxidant and anti-inflammatory abilities, the antiplatelet, anti-apoptotic and antihypertensive properties, the ability to improve endothelial function, the metabolic profile and ventricular remodeling, reduction of arterial stiffness as well as reduction of size of atherosclerotic plaque. Lycopene exerts favorable effects in patients with subclinical atherosclerosis, metabolic syndrome, hypertension, peripheral vascular disease, and several other cardiovascular disorders, but sometimes conflicting results were obtained. Clearly, more and better-designed studies will be necessary to improve our understanding of the positive effects of lycopene on vascular health and to elucidate the involved mechanisms on a molecular level.

Future cardiovascular disease prevention strategies might include lycopene-enriched products, lycopene supplementation and new combinations including lycopene. Future studies focused on dietary lycopene and its synergistic effects with other dietary components in different study populations, with elevated cardiovascular risk, are highly warranted and might enable development of functional foods useful in prevention and complementary treatment of cardiovascular disorders.

## Author contributions

IM is the author of the first draft of the manuscript. DS, AC, CM, JH, and AA contributed toward revising the paper and agree to be accountable for all aspects of the work. All authors agreed on the finally submitted version of the manuscript.

### Conflict of interest statement

The authors declare that the research was conducted in the absence of any commercial or financial relationships that could be construed as a potential conflict of interest.
